# Nutritional status and association of demographic characteristics with malnutrition among children less than 24 months in Kwale County, Kenya

**DOI:** 10.11604/pamj.2017.28.265.12703

**Published:** 2017-11-24

**Authors:** Morris Ndemwa, Sheru Wanyua, Satoshi Kaneko, Mohammed Karama, Makokha Anselimo

**Affiliations:** 1 Jomo Kenyatta University of Agriculture and Technology, Nairobi, Kenya; 2 Nagasaki University Institute of Tropical Medicine, Nairobi, Kenya; 3 African Population Health Research Center, Nairobi, Kenya; 4 Department of Eco-Epidemiology, Institute of Tropical Medicine, Nagasaki University, Japan; 5 Center for Public Health Research, Kenya Medical Research Institute (KEMRI) Nairobi, Kenya

**Keywords:** Nutritional status, stunting, wasting, underweight, malnutrition

## Abstract

**Introduction:**

Malnutrition is an underlying cause of mortality in about half of the cases that occur among children less than five years in developing countries. In Africa including Kenya, this problem may be exacerbated by socio-demographic and socio-economic factors. This study aimed at determining nutritional status and association of demographic characteristics with malnutrition among children aged 1 day to 24 months in Kwale County, Kenya.

**Methods:**

A cross-sectional study was done in Mwaluphamba Location, Kwale County, Kenya. Data was collected using a semi-structured questionnaire and administered to 380 randomly selected mothers who had children under the age of two years. Nutrition status was determined using anthropometric measurements. Data was analyzed using descriptive statistics and associations were determined by univariate logistic regression.

**Results:**

Malnutrition prevalence for children in Kwale was high with 29.2% of the children being stunted and 13.4% being severely stunted. Underweight prevalence was at 20.8% of whom 9.5% were severely underweight. The global acute malnutrition rate was 18.9%. Stunting differed significantly between sex (males 35.1% compared to females 21.7%; p = 0.005). Significant differences were also observed in stunting and underweight due to age (p < 0.005).

**Conclusion:**

The prevalence of stunting, underweight and global acute malnutrition rates was high among the children. Male children were associated with a significantly higher prevalence of stunting than the females. The prevalence of underweight and stunting significantly increased with increasing age.

## Introduction

Malnutrition in children is among the leading health problems that contribute to the high child morbidity and mortality in the developing world including Kenya. Globally, child malnutrition declined in the 1990s', but in Africa the number of malnourished children increased from 26 million to 32 million from 1990 to 2000. Additionally, 25% of all children under five years old were underweight by 2000, indicating little change [1]. Currently out of an estimated total of 670 million children under the age of 5 years, 160 million are stunted, 50 million are wasted while 41 million are overweight [2]. State of the world children report by UNICEF (2016) indicates that the malnutrition rate is still high in the world standing at 24% stunting, 14% underweight and 8% wasting for children under five. In Kenya, the stunting rate of these children stands at 26%, it is higher than the average world rate. Inadequate intake of energy and micronutrients by children leads to protein-energy malnutrition and micro-nutrient deficiencies. About 200 million children under 5 years of age in developing countries experience short growths due to factors like, poverty, poor health, poor nutrition and poor nurture habits among others [3, 4]. Malnutrition among children may manifest as low height-for-age (stunting) and is a factor in impairment of human development growth rate. In children, it refers to those that fall below 2 standard deviations from the median height-for-age based on the World Health Organization (WHO) reference standards. On the other hand, underweight is low weight-for-age below, 2 standard deviation from the median weight-for-age. Wasting, is described as low weight-for-height, a measure below 2 standard deviation from the median weight-for-height standard [5]. Stunting is an indicator of early chronic undernutrition, while wasting is an indicator for acute undernutrition. Underweight, is overall indicator of the combination of both stunting and wasting elements in children [6]. The likelihood of having severe malnourishment in children is related to social economic and social demographic characteristics. For instance, children with single parents or parents who have lower education level are more likely to be malnourished than children with both parents and parents who have a higher education level [7].

Stunting affects women adult height, which in turn affects their reproductive health, survival of their children. In men, economic productivity is negatively affected as a result of stunting in childhood. Secondly, stunting reflects damage that affects, in some cases, irreparably health and development over the long term [8]. Stunting is an attributing risk factor to low birth weight (LBW) in children [9]. Nutritional intervention needs to be implemented early during the “window of opportunity” period that includes the pre-conception period, pregnancy, lactation and the first 2-3 years of life. It is difficult to reverse after 36 months of age. Sustainable development goals (2 and 3) of improving nutrition and ensuring healthy lives and well-being for all ages in line with the earlier Millennium Development Goals of achieving universal primary education, eradicating poverty, reducing child mortality and improving maternal health can contribute to reducing stunting [8, 10-12]. Adverse climatic conditions worsen the problem of malnutrition. Unfortunately, most governments have been unsuccessful in reducing malnutrition due to lack of appropriate policies that are effective in reducing malnutrition [13]. Findings from the Nutritional Collaborative Research Support Program (CRSP) in Kenya, Mexico and Egypt demonstrate how the etiology of the early onset of stunting varies among populations of varying biological, environmental and cultural circumstances [12, 14-16]. According to the Kenya Demographic and Health Survey (KDHS) of 2009, Coast Province had a higher rate of malnutrition among children under 5 years of age than the national average, with a stunting level of about 39%, the second highest in the country. The 2014 Kenya Demographic and Health Survey (KDHS) shows that this has reduced over the years and was reported to be at 29.7%, but was still the second highest in the country [17, 18]. This study aimed at determining nutritional status and association of demographic characteristics with malnutrition among children less than 24 months in Kwale County, Kenya.

## Methods

The study was conducted in Mwaluphamba Location, using a descriptive cross-sectional design that applied quantitative methods to elucidate nutritional status and association of demographic characteristics with malnutrition among children aged 1 day to 24 months in Kwale County, Kenya. The area is within Kwale Health and Demographic Surveillance System which was established in July 2010 and covered 7,617 households and 51,000 inhabitants, in an area of 384.9 Km^2^ as of July 1, 20^11^ [19]. The site was chosen because of the high prevalence rate of stunting among children in the Coastal part of Kenya [18]. The main economic activities of the people in Kwale are mixed farming, and informal employment. Mwaluphamba Location was purposively chosen for the study because it had the highest population of under-fives (5283). The study population comprised children less than 24 months of age and their mothers/caregivers within the HDSS study site of Mwaluphamba Location. The respondents were the mothers/caregivers. The sampling frame was the population obtained from the Nagasaki University Institute of Tropical Medicine-Kenya Medical Research Institute (NUITM-KEMRI) database which contains demographic and other data collected regularly from the Kwale HDSS. The selection of mother/caregiver-child pairs was done by simple random sampling from a list of the HDSS study. Random computer generated numbers were used to select 366 children. The sample size was adjusted to 385 mother/child pairs (5% addition to the sample size calculated) to replace any refusals. The sample size for the study was derived from Cochran's method [20] of sample size determination with a 95% confidence interval and a sampling error of 5%. Subjects eligible for the study were mothers/caregivers with children aged 1 day to 24 months and must have lived in the area for the previous 6 months and consented for the study. Mothers who were observed to be unwell/sick/ill were excluded from the study. A semi-structured questionnaire was pre-tested outside the study area and revised for consistency. Local interviewers were trained to administer the questionnaire and to collect data. Collected data included; basic demographics and social economic factors as well as anthropometric measurements which were recorded using standard anthropometric techniques. Determination of nutritional status was done using anthropometry.

Children 0-6 months were weighed using Slovakia Aid (Tramtron, Slovakia) scale and the weight was recorded to the nearest 0.1 kg. Older children who could not stand were measured using baby trousers and a portable electronic scale to measure the weight. They were first hung from suitable secure points such as trees and doorframes and the scale adjusted to zero with an empty weighing pant suspended from the lower hook. The minimally clothed child was then placed in the weighing pant and suspended on the weighing scale to hang freely and weight recorded to the nearest 0.1 kg. For children who could stand, weight was taken using an electronic Tanita THD-650 scale (Tanita, Japan). The scales were placed on a flat surface and set at zero. The mothers were lightly dressed with no shoes and heavy clothing. Weight was recorded to the nearest 0.1 kg. Height measurements were done using height boards. Height for children below 12 months was measured in supine position while that of children aged 12 months and older was measured in a recumbent position. To measure height, the height board were placed upright on a flat surface leaning against a secure point such as a wall, tree or supported by one team member standing behind it. The barefooted child, with no head ornaments or high hair styles, was then made to stand against the height board looking straight ahead with the back of the head, shoulders, buttocks, calves and heels touching the upright board. The cursor was then lowered to gently and firmly press on any hair and height measurement taken to the nearest 0.1cm. The heights of mothers were taken using adult height boards. These were placed on a flat surface leaning on secure points and barefooted subjects requested to stand upright (with the back of the head, shoulders, buttocks, calves and heels touching the upright board). Looking straight ahead and without high hair styles, the cursor was brought down to gently and firmly presses on the hair on the crown of the head and height taken to the nearest 0.1cm. The MUAC of children was recorded using child MUAC tapes. The MUAC was taken on the left arm. The measure of the length of the child's upper arm, between the bone at the top of the shoulder and the elbow bone (the child's arm ought to be bent) was taken. Middle of the child's upper arm was marked with a pen. The child's arm was then relaxed, falling alongside its body. MUAC tape was wrapped around the child's arm, such that all of it was in contact with the child's skin. It was neither too tight, nor too loose. The MUAC was then read and recorded to the nearest 0.1cm.

Bilateral oedema was determined by applying thumb pressure on the top part of both feet for 3 seconds. If pitting occurred on both feet upon release of the fingers, the child was recorded as having nutritional oedema, denoting severe malnutrition and referred to the nearest health facility for management. Calibration of weighing scales with known weights was done every day for quality assurance purposes. In addition, pretesting of the questionnaire was done on a sub sample of 30 mother-child pairs. Permission to conduct the study was given by the Kenya Medical Research Institute KEMRI Scientific Steering Committee (SSC) and the Ethical Review Committee (ERC) SSC NO. 2534. Informed consent was obtained from the participants and those who agreed to participate were requested to sign the informed consent forms. For the illiterate, consent was given in form of finger print before commencing the study. Confidentiality was assured and data was anonymized. Data was entered in Microsoft Access 2013 (Microsoft, WA, USA) and then imported to Stata Statistical software (Version 13.1: Stata Corporation, TX USA) for cleaning and data analysis. Comparisons were done for the anthropometric measurements with Z scores established by WHO. Moderate stunting (low height-for-age) in children, was categorized to children that had a score of between negative two and negative three standard deviation below (-2SD/Z to-3SD/Z) of the median height-for-age established by the WHO. Severe stunting was classified to children with a Z-score of -3SD three standard deviation and below from the mean established standard value. Moderate low weight-for-age (underweight) in children was obtained as a measure for weight-for-age who were between negative two and negative three standard deviation score (-2SD/Z to -3SD/Z) from the median weight-for-age. Severe underweight was classified to children with a Z-score of negative three standard deviation (-3Z) and below from the mean standards while children with a Z-score of between negative two and negative three standard deviation (-2SD/Z to -3SD/Z) were categorized as moderately underweight.

Wasting, weight-for-height (WHZ) was described as low weight-for-height a measure below (-2Z) two standard deviation from the median weight-for-height standard. Severe wasting was classified to children with a Z-score of three standard deviation and below from the mean standards of WHZ). MUAC was recorded for children aged between 6 months and 24 months. Classification of MUAC was on the basis of the WHO established cutoffs of less than 110mm (11.0cm), for Severe Acute Malnutrition (SAM). Children with a MUAC between 110mm (11.0cm) and 125mm (12.5cm), were grouped to Moderate Acute Malnutrition (MAM). MUAC of between 125mm (12.5cm) and 135mm (13.5cm), indicated that the children were at risk of acute malnutrition while MUAC over 135mm (13.5cm), was showing good nourishment. Calculations of z-scores was done using the formula: (Measured value-Average value in reference population)/Standard deviation of the reference population. Body Mass Index (BMI) for non-pregnant women was computed using the formula: BMI (Body Mass Index) = weight in kilograms/height in meters squared. The BMI values for mothers/caregivers were categorized as underweight if their BMI was below 18.5, normal or healthy if their BMI ranged between 18.5-24.9, overweight if BMI ranged 25.0-29.9 and obese if the BMI was 30.0 and above. Descriptive statistics of the data were determined by frequencies, percentages, range, mean ± standard deviation, Association between stunting and explanatory variables were determined by univariate logistic regression analyses. Multiple logistic regression analysis was used to determine association of numerous factors on the outcome variables-stunting, under-weight and wasting.

## Results

**Figure 1:**
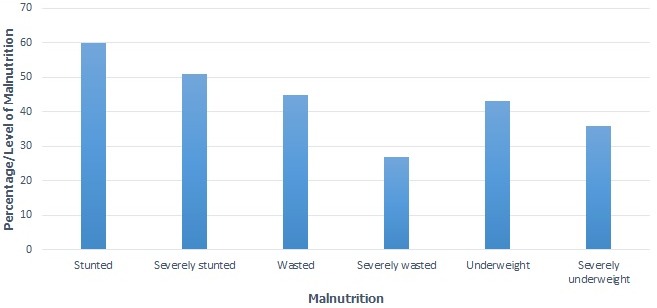
Summary of malnutrition for children (day 1 to 24 months) in Kwale HDSS (n = 380

**Figure 2:**
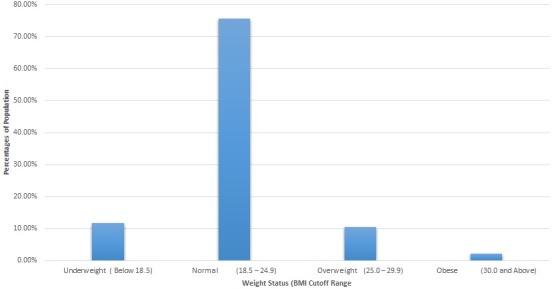
Summary of mothers/caregivers nutritional status

Five mother-child pairs were excluded from the research study because of the following reasons: two mother- child pairs migrated outside from the HDSS area; two mothers refused to give consent to participate in the study and one child was severely sick. The final number of mother child pairs that participated in the study were 380. The final number of children who met the set inclusion criteria were 380 in total of whom 214 (56.3%) were boys and 166 (43.7%) were girls. The median age (months) for the children was 9 months with IQR of 10 (4-14). Median age (months) for boys was 9 and Interquartile Range (IQR) of 11(4-15) while for the girls it was median of 8 and IQR of 11 (3-14). Children in the study were categorized into 3 age-groups; ≤ 5 months who were 125 (32.9%), 6-11 months were 114(30%) and 12-24 months were 141(37.1%). There were 380 mothers/caretakers for the 380 children with a mean age of 28.6 years and a range of 15-61 years. More than half of the mothers (63%) were below 30 years of age, with the highest proportion (28.2%) being between 25-29 years. The mothers/caregivers were classified into eight age categories: 15-19 years 47(12.4%), 20-24 years 85(22.4%), 25-29 years 107(28.2%), 30-34 years 67(17.6%), 35-39 years 48(12.6%), 40-44 years 18(4.7%), 45-49 years 3(0.8%) and 50+ years 5(1.3%). Majority of mothers/caregivers (62.4%) had achieved primary education while 29.5% had no formal education. Overall summary of social demographic characteristics is shown in Table 1. Prevalence of malnutrition for children in Kwale was considerably high with 29.2% being stunted, of whom 13.4% were severely stunted. About one fifth, (20.8%) were underweight, with 9.5% being severely underweight. The proportion of wasting was 18.9% for these children, and of whom 7.1% were severely wasted as shown in Figure 1. Overall prevalence of malnutrition for mothers/caregivers, including both over-nutrition and under nutrition was 24.4% (Figure 2). Over-nutrition is the condition where intake of food is in excess of dietary energy requirements, resulting in overweight and/or obesity while Under-nutrition is the result of food intake that is insufficient to meet dietary energy and nutrient requirements. The prevalence of stunting among male children (35.1%) was significantly higher (p ≤ 0.05) than that of female children (21.7%). Stunting increased significantly (p ≤ 0.05) with increase in age-group categories for children at 0-5(12.8%), 6-11(29.0%) and 12-24(44.0%) as shown in Table 2, Table 2 (suite).

**Table 1:** Summary of social demographic characteristics of children and their mothers/caregiver

Characteristics	Total n=380	Percentage (%)
**Gender**		
Male	214	56.3
Female	166	43.7
**Age (Months) Children**		
0 - 5	125	32.9
6 - 11	114	30.0
12 - 24	141	37.1
Age (Years) Mother	380	
15 - 19	47	12.4
20 - 24	85	22.4
25 - 29	107	28.2
30 - 34	67	17.6
35 - 39	48	12.6
40 - 44	18	4.7
45 - 49	3	0.8
50 +	5	1.3
**Mothers/Caretakers Employment Status**		
Formal employment	351	92.4
Non-Formal employment	29	7.6
**Highest Level of Education**		
No formal education	112	29.5
Primary	237	62.4
Secondary	20	5.3
College/university	11	2.9

**Table 2:** Association of child stunting and underweight with social demographic characteristics using chi-square and multiple logistic regression (N=380)

Child Stunting by chi-square and multiple logistic regression (N=380)						Underweight by chi-square and multiple logistic regression (N=380)				
Social demographic characteristics	Stunting Association by Chi-square and Logistic Regression					Underweight Association by Chi-square and Logistic Regression				
Gender child	No n (%)	Yes n (%)	Total	P-Value	ADD OR(CI)P-Value	No n (%)	Yes n (%)	Total	P-Value	ADD OR(CI)P-Value
Female	130 (78.31)	36 (21.69)	166 (43.68)	**0.005**	**R**	135 (81.33)	31 (18.67)	166 (53.68)	0.371	**R**
Male	139 (64.95)	75 (35.05)	214 (56.32)		1.95 (1.2 - 3.1) 0.005	166 (77.57)	48 (22.43)	214 (56.32)		1.26 (0.76-2.09) 0.37
**Age-child (months)**										
0-5	109 (87.2)	16 (12.8)	125 (32.89)	**< 0.001**	**R**	110 (88.0)	15 (12.0)	125 (32.89)	0.005	**R**
6-11	81 (71.05)	33 (28.95)	114 (30.0)		2.78 (1.4 - 5.39) 0.003	90 (78.95)	24 (21.05)	114 (30.0)		1.96 (0.97 - 3.95) 0.06
12-24	79 (56.03)	62 (43.97)	141 (37.11)		5.35 (2.9-9.95) < 0.001	101 (71.63)	40 (28.37)	141 (37.11)		2.9 (1.51- 5.57) 0.1
**Age Mother (Years)**										
15-19	37 (78.72)	10 (21.28)	47 (12.37)	0.375	**R**	40 (85.11)	7 (14.89)	47 (12.37)	0.697	**R**
20-24	64 (75.29)	21 (24.71)	85 (22.37)		1.21 (0.5 - 2.85) 0.66	71 (83.53)	14 (16.47)	85 (22.37)		1.13 (0.42 -3.02) 0.81
25-29	74 (69.16)	33 (30.84)	107 (28.16)		1.65 (0.7 - 3.71) 0.23	81 (75.7)	26 (24.3)	107 (28.16)		1.83 (0.73 - 4.59) 0.2
30-34	49 (73.13)	18 (26.87)	67 (17.63)		1.36 (0.6 - 3.29) 0.5	53 (79.1)	14 (20.9)	67 (17.63)		1.51 (0.56 - 4.09) 0.42
35-39	29 (60.42)	19 (39.58)	48 (12.63)		2.42 (1 - 6) 0.06	38 (79.17)	10 (20.83)	48 (12.63)		1.5 (0.52 - 4.35) 0.45
40-44	12 (66.67)	6 (33.33)	18 (4.74)		1.85 (0.6 - 6.16) 0.32	13 (72.22)	5 (27.78)	18 (4.74)		2.2 (0.59 - 8.12) 0.24
45-49	2 (66.67)	1 (33.33)	3 (0.79)		1.85 (0.2 - 22.5) 0.63	2 (66.67)	1 (33.33)	3 (0.79)		2.86 (0.23 - 35.91) 0.42
50+	2 (40.00)	3 (60.00)	5 (1.31)		5.55 (0.8 - 37.9) 0.008	3 (60.0)	2 (40.0)	5 (1.32)		3.81 (0.54 - 27.08) 0.18
**Mothers/Caretakers Education Level**										
College/university	10 (87.5)	1 (12.5)	8 (2.11)		**R**	10 (87.5)	1 (12.5)	11 (2.11)		**R**
Secondary	15 (75)	5 (25)	20 (5.26)		2.33 (0.2 - 23.9) 0.48	17 (85.0)	3 (15.0)	20 (5.26)		1.24 (0.11 - 14.01) 0.87
Primary	167 (70.46)	70 (29.54)	237 (62.37)		2.93 (0.4 - 24.3) 0.32	192 (81.01)	45 (18.99)	237 (62.37)		1.64 (0.2 - 13.67) 0.65
No formal education	77 (68.75)	35 (31.25)	112 (29.47)	0.604	3.18 (0.4 - 26.8) 0.29	82 (73.21)	30 (26.79)	112 (29.47)	0.35	2.56 (0.3 - 21.69) 0.39
**Mothers/CaregiversEmployment Status**										
Formal employment	244 (69.52)	107 (30.48)	351 (92.37)	0.057	**R**	24 (82.76)	5 (17.24)	29 (7.63)	0.624	**R**
Non-formalemployment	25 (86.21)	4 (13.79)	29 (7.63)		0.36 (0.1 - 1.07) 0.07	277 (78.92)	74 (21.08)	351 (92.37)		0.78 (0.29 - 2.11) 0.63

**Table 2:** (B) Association of child wasting with social demographic characteristics using chi-square and multiple logistic regression (N=380)

	Child Wasting by chi-square and multiple logistic regression (N=380)				
Social demographic characteristics	Wasting Association by Chi-square and Logistic Regression				
Gender child	No n (%)	Yes n (%)	Total	P-Value	ADD OR(CI) P-Value
Female	130 (78.31)	36 (21.69)	166 (43.68)		R
Male	178 (83.18)	36 (16.82)	214 (56.32)	0.23	0.73(0.44 -1.22) 0.231
**Age-child (months)**					
0-5	102 (81.6)	23 (18.4)	125 (32.89)	0.413	R
6-11	88 (77.19)	26 (22.81)	114 (30.0)		1.31 (0.7 - 2.46) 0.4
12-24	118 (83.69)	23 (16.31)	141 (37.11)		0.86 (0.46 - 1.63) 0.65
**Age Mother (Years)**					
15-19	34 (72.34)	13 (27.66)	47 (12.37)	0.508	R
20-24	68 (80.0)	17 (20.0)	85 (22.37)		0.65 (0.28 - 1.5) 0.32
25-29	88 (82.24)	19 (17.76)	107 (28.16)		0.56 (0.25 - 1.27) 0.17
30-34	57 (85.07)	10 (14.93)	67 (17.63)		0.46 (0.18 - 1.16) 0.1
35-39	41 (85.42)	7 (14.58)	48 (12.63)		0.45 (0.16 - 1.24) 0.12
40-44	13 (72.22)	5 (27.78)	18 (4.74)		1.01 (0.3 - 3.38) 0.99
45-49	2 (66.67)	1 (33.33)	3 (0.79)		1.31 (0.11 - 15.68) 0.83
50+	5 (100.0)	0 (0.00)	5 (1.32)		0.78 (0.29 -2.11) 0.63
**Mothers/Caretakers Education Level**					
College/university	8 (62.5)	3 (37.5)	11 (2.90)		R
Secondary	18 (90.0)	2 (10.0)	20 (5.26)		0.19 (0.02 - 1.43) 0.11
Primary	197 (83.12)	40 (16.88)	237 (62.37)		0.34 (0.08 - 1.47) 0.15
No formal education	85 (75.89)	27 (24.11)	112 (29.47)	0.189	0.53 (0.12 - 2.36) 0.41
**Mothers/CaregiversEmployment Status**					
Formal employment	23 (79.31)	6 (20.69)	29 (7.63)	0.803	R
Non-formal employment	285 (81.2)	66 (18.8)	351 (92.37)		1.31 (0.11- 15.68) 0.83

Differences observed with regard to underweight (males 22.4%, females 18.7%; p = 0.37) were not significant between the sexes while underweight between age-groups was statistically significant (0-5 months 12.0%, 6-11 months 21.1% and 12-24 months 28.4%; p = 0.005) as shown in Table 2, Table 2 (suite). There was a higher prevalence for wasting among female children in comparison to the males, but this was not statistically significant (males 16.8%, females 21.7%; p = 0.41). There was evidence of wasting but it was not statistically significant between the sexes as shown in Table 2, Table 2 (suite) below. There was no significant difference in the prevalence of child stunting due to mothers age, education and employment status. Prevalence of stunting reduced with increase in the level of education of mothers/caretakers. High prevalence of stunting is seen at 31.3% for mothers/caretakers who had no formal education and is lowest at 12.5% for those who have attained college/university level of education but this had no statistical association p = 0.60. Male children were 1.95 more likely to be stunted than female children with p < 0.05. Children at 6-11 months age category had a 2.78 adjusted odds ratio with a statistical significance of p < 0.003 while children at 12-24 months age 5.35 times likely to be stunted with a p value of p < 0.001 as shown in Table 2, Table 2 (suite). Caretakers/mothers who were employed or working seem to have children who had high levels of stunting (30.5%) compared to those that were not working at 13.79% even though this was not statistically significant p = 0.06. On assessment of malnutrition using MUAC, a proportion of 15.7% (40) of the total children 6 months and older (255) were malnourished, with a MUAC of less than 12.5cm. Moderate acute malnutrition was 14.9% while severe acute malnutrition was 0.8%. The mothers mean Body Mass Index (BMI) was 21.6 and range of 14.5-36.5. Figure 2 shows results of BMI distribution for mothers/caretakers with overall, 24.5% malnutrition of whom 2.1% were obese, 10.5% overweight and 11.8% were underweight. The Obese class of women, 37.5% was highest for 30-34 and 35-39 age-groups respectively. Prevalence of overweight and underweight was highest at the 25-29 age-group with 35% and 31.1%, respectively.

## Discussion

In this study, the prevalence of malnutrition among the children was relatively high, with stunting at 29.2%, underweight 20.8% and wasting 19.0%. The stunting rate is above the national mean of 26% for children under five [17]. This high malnutrition prevalence conforms to the observation that malnutrition has been a leading cause of high risk of morbidity and mortality in Africa and especially in sub-Saharan Africa. The risk is exacerbated by other factors including tropical diseases, health inequities, social economic status (SES) and others [21]. According to World Health Assembly (WHA), Stunting is a critical impediment to human development worldwide affecting children under the age of 5 years in millions [22, 23]. The proportion of male children who were stunted was significantly higher than that of girls. This is similar to the observations in other studies [24]. It may be due to differences in in feeding and care practices of the two genders [24]. Statistically significant differences were observed in stunting between age-groups which was more than double as children grow older from 12.8% in 0-5 months to 29.0% in 6-11 months then further to 44.0% in 12-24 months age-group. These differences can be explained by; the time mothers introduce complementary foods, type of foods used for complementary feeding, feeding practices including the number of meals taken in a day and the care of children. Care level declines as children grow older leading to growth faltering. Similarly, weight for age (underweight) seems to increase significantly as the children grow older. These results are in agreement with other studies where child age, mothers' education status, marital status and livestock ownership are main predictors of malnutrition in children under the age of 5 years in Ethiopia [23, 25]. In this study, based on MUAC, 15.7% of the children between 6-24 months suffered global acute malnutrition, of whom 0.8% had severe acute malnutrition. Severe acute malnutrition often comes with complications although can be managed using WHO guidelines. These guidelines have been shown to reduce mortality associated with SAM by less than 5% [26, 27]. Mothers' literacy level is a predictor of children nutrition status [28]. In this study, mothers' education level was proportionally associated with underweight in children. Study results suggest that proportion level of malnutrition (underweight) in children decreased as mothers education level increased. This can be explained by the care given by mothers to children gradually increasing as the level of education increased. The outcome of this difference was not statistically significant in our study. Mothers' employment status did not have statistically significant differences among different employment categories. Working mothers are more often away from home than non-working mothers or stay home mothers. Time and care given to children is therefore not comparable. Our study gives a higher percentage of underweight children belonging to working mothers than children belonging to non-working mothers. Assessment by BMI showed that one quarter (24.5%) of mothers were malnourished. Association of low mothers' BMI, occupation, education, low birthweight among others are factors that are significant predictors of child under-nutrition [29, 30].

## Conclusion

The study showed a relatively high prevalence of malnutrition among the children. The stunting, underweight and wasting rates were above the national means. Male children were associated with a significantly higher prevalence of stunting than the female children. Male children were more likely to be stunted than female children. The prevalence of underweight and stunting significantly increased with increasing age. Based on these results, more efforts and study programs are needed to address the problem of malnutrition among children. There should also be focus on practices that lead to malnutrition differences between gender and age among children.

### What is known about this topic

- Childhood undernutrition is declining in Kenya generally, but remains significantly higher is some parts of the country;
- Prevalence of malnutrition is higher in male than in female children.

### What this study adds

- Malnutrition differences between genders in children is depended on childcare and practices;
- Social demographic characteristics are determinants of malnutrition differences between gender and age among children in the marginalized regions.

## Competing interests

The authors declare no competing interests.
